# Common functional alterations identified in blood transcriptome of autoimmune cholestatic liver and inflammatory bowel diseases

**DOI:** 10.1038/s41598-019-43699-1

**Published:** 2019-05-10

**Authors:** Jerzy Ostrowski, Krzysztof Goryca, Izabella Lazowska, Agnieszka Rogowska, Agnieszka Paziewska, Michalina Dabrowska, Filip Ambrozkiewicz, Jakub Karczmarski, Aneta Balabas, Anna Kluska, Magdalena Piatkowska, Natalia Zeber-Lubecka, Maria Kulecka, Andrzej Habior, Michal Mikula, The Polish PBC study Group, Bozena Walewska-Zielecka, Marek Krawczyk, Halina Cichoz-Lach, Piotr Milkiewicz, Agnieszka Kowalik, Krzysztof Mucha, Joanna Raczynska, Joanna Musialik, Grzegorz Boryczka, Michal Wasilewicz, Irena Ciecko-Michalska, Malgorzata Ferenc, Maria Janiak, Alina Kanikowska, Rafal Stankiewicz, Marek Hartleb, Tomasz Mach, Marian Grzymislawski, Joanna Raszeja-Wyszomirska, Ewa Wunsch, Tomasz Bobinski, The Polish IBD study Group, Jaroslaw Kierkus, Piotr Socha, Michal Lodyga, Maria Klopocka, Barbara Iwanczak, Katarzyna Bak-Drabik, Jaroslaw Walkowiak, Piotr Radwan, Urszula Grzybowska-Chlebowczyk, Bartosz Korczowski, Teresa Starzynska

**Affiliations:** 1 Department of Genetics, Maria Sklodowska-Curie Institute – Oncology Centre, Warsaw, 02-781 Poland; 2 Department of Gastroenterology and Hepatology, Medical Center for Postgraduate Education, Warsaw, 02-781 Poland; 3 Department of Pediatric Gastroenterology and Nutrition, Medical University of Warsaw, Warsaw, 02-091 Poland; 4 Department of Public Health, Faculty of Health Sciences, Medical University of Warsaw, Warsaw, Poland; 5 Department of General, Transplant and Liver Surgery, Medical University of Warsaw, Warsaw, Poland; 6 Department of Gastroenterology, Medical University, Lublin, Poland; 7 Department of General, Liver and Internal Medicine Unit, Transplant and Liver Surgery, Medical University of Warsaw, Warsaw, Poland; 8 Department of Clinical and Molecular Biochemistry, Pomeranian Medical University, Szczecin, Poland; 9 Department of Immunology, Transplantology and Internal Medicine, Medical University of Warsaw, Warsaw, Poland; 10 Institute of Biochemistry and Biophysics, Polish Academy of Sciences, Warsaw, Poland; 11 Department of Gastroenterology and Hepatology, Medical University of Silesia, Katowice, Poland; 12 Department of Gastroenterology and Infectious Diseases, Collegium Medicum Jagiellonian University, Krakow, Poland; 13 Department of Gastroenterology, Provincial Hospital, Olsztyn, Poland; 14 Department of Gastroenterology and Hepatology, Medical University of Gdansk, Gdansk, Poland; 15 Department of Internal and Metabolic Diseases and Dietetics, Poznan University of Medical Sciences, Poznan, Poland; 16 Department of Gastroenterology, Provincial Hospital, Ostroleka, Poland; 17 Department of Gastroenterology, Hepatology and Feeding Disorders, Children’s Memorial Health Institute, Warsaw, 04-730 Poland; 18 Department of Internal Medicine and Gastroenterology with IBD Subdivision, Central Clinical Hospital of the Ministry of the Interior, Warsaw, 02-507 Poland; 19 Vascular Diseases and Internal Medicine, Nicolaus Copernicus University in Torun, Collegium Medicum, Bydgoszcz, 85-067 Poland; 20 Department of Pediatrics, Gastroenterology and Nutrition, Wroclaw Medical University, Wroclaw, 50-367 Poland; 21 Department of Pediatrics, School of Medicine with the Division of Dentistry in Zabrze, Medical University of Silesia, Katowice, 40-752 Poland; 22 Department of Pediatric Gastroenterology &Metabolic Diseases, Poznan University of Medical Sciences, Poznan, 61-701 Poland; 23 Department of Gastroenterology, Medical University of Lublin, Lublin, 20-059 Poland; 24 Department of Pediatrics, School of Medicine in Katowice, Medical University of Silesia, Katowice, 40-752 Poland; 25 Medical College, University of Rzeszow, Rzeszow, 35-959 Poland; 26 Department of Gastroenterology, Pomeranian Medical University, Szczecin, 70-204 Poland

**Keywords:** Primary biliary cirrhosis, Primary sclerosing cholangitis, Crohn's disease, Ulcerative colitis

## Abstract

Primary biliary cholangitis (PBC), primary sclerosing cholangitis (PSC), and inflammatory bowel diseases (IBDs), including Crohn’s disease (CD) and ulcerative colitis (UC), are heterogeneous chronic autoimmune diseases that may share underlying pathogenic mechanisms. Herein, we compared simultaneously analyzed blood transcriptomes from patients with PBC, PSC, and IBD. Microarray-based measurements were conducted using RNA isolated from whole blood samples from 90, 45, 95 and 93 patients with PBC, PSC, CD, and UC, respectively, and 47 healthy controls. Expression levels of selected transcripts were analyzed by quantitative reverse-transcribed PCR using an independent cohort of 292, 71 and 727 patients with PBC, PSC, and IBD, respectively. Of 4026, 2650 and 4967 probe sets differentially expressed (adjusted p-value < 0.05) in samples from patients with PBC, PSC, and IBD, respectively, compared with healthy controls, 1946 were common to all three comparisons. Functional analyses indicated that most terms enriched for genes differentially expressed in PBC, PSC, and IBD patients compared with healthy controls were related to mitochondrial function, the vesicle endomembrane system, and GTPase-mediated processes. This study indicates that microarray-based profiling of blood gene expression supports research into the molecular mechanisms underlying disease, rather than being useful for selection of diagnostic biomarkers for use in clinical practice.

## Introduction

Primary biliary cholangitis (PBC), primary sclerosing cholangitis (PSC), and inflammatory bowel diseases (IBDs), including Crohn’s disease (CD) and ulcerative colitis (UC), are heterogeneous chronic autoimmune diseases with genetic, immunologic, and environmental components. Genetic risk factors for these conditions are primarily non-protein-coding single nucleotide polymorphisms with similar small effect sizes^1–3^.

PBC is characterized by lymphoplasmacytic infiltration around the interlobular ducts of the liver, resulting in progressive immune-mediated destruction of interlobular biliary ductules associated with a classical feature of autoimmune conditions, antimitochondrial antibodies. PSC manifests as cholangiocytic injuries associated with nonspecific inflammation. In both of these cholangiopathies, progressive fibrous obliteration of the intrahepatic and extrahepatic biliary tree results in chronic cholestasis leading to liver cirrhosis^1,4–6^. PBC occurs more frequently in women than men and primarily in middle age, with prevalence rates ranging from 40 to 400 patients per million and an incidence range of 0.7 to 49 per million^7–10^. PSC affects 9 to 13 patients per million annually with a male-to-female ratio of 2:1^11^. Up to 80% of PSC cases are associated with IBD, while PSC is present in 3–8% of all patients with UC and 1–3% of patients with CD^12,13^.

IBDs result from multiple intestinal immunopathological processes, in which Th17 cells have a central role, in response to host intestinal microflora that induce the initiation and maintenance of intestinal inflammation^14^. Of the two major types of IBD, UC is characterized by inflammation extending continuously from the rectum along the entire colon, while in CD the inflammatory response is typically localized to the distal small intestine and colon. In UC, inflammation is confined to the mucosal surface of the colon, while in CD it is transmural. IBD onset can occur from early childhood to beyond the sixth decade of life, with childhood-onset IBD representing 10–25% of all cases^15^. Moreover, while PBC and PSC are progressive disorders, IBDs typically present as repeated cycles of relapse and remission of intestinal inflammation.

Blood comes into contact with the cells, tissues, and organs of the entire organism and constitutes a primary aspect of the immune defense system. Hence, it is not surprising that gene expression changes in white blood cells (WBCs) are associated with a wide range of pathological conditions. Blood can be considered as a surrogate for traditional tissue specimens employed for clinical diagnosis, and analyses of WBC expression profiles provide a non-invasive method that can be used to support investigations of both the molecular mechanisms underlying disease and medical practice^16^. Although the fibrous cholangiopathies, PBC and PSC, and nonspecific IBDs, UC and CD, exhibit significant differences in their clinical presentation, chronic inflammation and dysregulated immune responses are common to both types of disorder. Consequently, similar risk factors may be implicated in their pathogenesis, particularly given the crosstalk between bile acids (BAs) and gut microbes^17^. Once the bile duct and intestinal defense systems become affected, inappropriate innate immune and inflammatory responses may contribute to disturbed antibacterial reactive oxygen species (ROS)-mediated and mitochondrial autophagy. Whether alterations similar to those in tissues directly affected by disease can be observed within WBCs remains open to question. While several previous studies uncovered alterations of WBC gene expression in IBDs^18–21^, no comparable investigations of patients with PBC or PSC have been reported to date.

The main aim of our study was to uncover possible pathomechanisms common for PBC, PSC, UC, and CD by analysis of blood-based transcriptomes simultaneously generated for all of them. Additionally, basing on microarray profiling we intended to identify their new biomarkers. However, although our study selected aberrations of cellular signaling and regulatory pathways shared across all of the studied disorders, we did not select genes which could be used in a diagnostic screening.

## Materials and Methods

### Ethics approval and consent to participate

The study was approved by the ethics committee (decision 46/PW/2011) of the Medical Center for Postgraduate Education, Warsaw, Poland. Informed consent was obtained from all subjects or, if subjects were under 18, from a parent and/or legal guardian. The study protocol conforms to the ethical guidelines of the 1975 Declaration of Helsinki.

### Study subjects

Patient cohorts included in this study comprised the following: 382 female patients with PBC, 331 of whom were antimitochondrial antibody positive; 116 (33 female and 83 male) patients with PSC; and 915 (486 females and 429 males) patients with IBD. Of patients with IBD, 488 (303 children) and 427 (246 children) were diagnosed with CD and UC, respectively. All enrolled patients and controls were Polish Caucasians. Most PSC patients were diagnosed with IBD: 12 with CD and 93 with UC. Diagnosis of PBC was based on standard clinical, biochemical, serological, and histological criteria, and PSC was diagnosed according to standard clinical, biochemical, cholangiographic, and (in some patients) histological criteria, according to the European Association for the Study of Liver (EASL)^14^. Before inclusion, all PBC patients and some PSC patients were treated with ursodeoxycholic acid; 20 PSC patients then underwent liver transplantation. IBDs were diagnosed using the Porto criteria, modified in accordance with the recommendations of the European Crohn’s and Colitis Organization (ECCO) for children, and according to ECCO guidelines for adults. The CD activity index (CDAI), the UC activity index (UCAI), and their pediatric versions (PCDAI/PUCAI) were determined to evaluate disease severity^22–24^. Before inclusion most IBD patients were given mesalazine, and in majority of them the blood samples were collected before additional medication regimes (immunosupressants, glucocorticoids, biologic therapy) were ordered. Blood samples from 184/82/253 healthy individuals served as controls for PBC/PSC/IBD, respectively. Mean AST/ALT for PBC patients was 3.4/3.1, respectively. Summaries of the main epidemiological variables for each group are presented in Table 1.

**Table 1:** Summary of the main epidemiological variables for the discovery and replication cohorts.

		PBC	PSC	IBDs				Controls
				Children		Adults		
				CD	UC	CD	UC	
Discovery cohorts	Females/Males	90/0	19/27	29/21	28/19	23/22	26/20	26/21
	Age; range	29–76	16–53	2–17	1–17	19–69	21–66	38–62
	Age; median	57	29	13	15	34	36	46
Replication cohorts	Females/Males	292/0	14/56	103/150	111/88	86/54	80/55	107/99
	Age; range	18–85	17–64	2–17	1–17	18–70	18–73	29–78
	Age; median	56	31	15	15	29	35	49

### RNA extraction

For RNA extraction, whole blood was collected and total RNA was isolated using the Tempus RNA Isolation Kit (Thermo Fisher Scientific), according to the manufacturer’s instructions. RNA quality and quantity were analyzed using a NanoDrop spectrophotometer, and samples with A260/A280 ratios of 1.8–2.1 were further assessed using an Agilent 2100 Bioanalyzer. Samples used for microarray analysis had RNA integrity numbers in the range 7.6–9.6.

### Gene expression microarray analysis

Whole-transcriptome profiling was performed by AROS Applied Biotechnology services, using an HT-12 v4 Expression BeadChip (Illumina, San Diego, CA, USA). The average bead signals from the chip were quantile normalized, with no background correction. All computations were performed using R 3.4.1 software with the Bioconductor extension^25^. Principal component analysis (PCA) was used for the initial quality inspection. 9 samples (3 PSC, 3 CU, 1 PBC, 1 CD and 1 control) were removed as outliers. Probe sets with expression detected (detection p-value < 0.05) in less than 5 samples were discarded. The remaining measurements were filtered according to the ratio of the range between the 10th and 90th percentile (IQR10) and the median normalized IQR10 (NIQR10). Only probes with NIQR10 values higher than the median NIQR10 for the whole set were selected for analysis. Genes showing differential expression were selected according to p-value determined by t-test (Welch’s variant) after correction for multiple hypothesis testing using the Benjamini–Hochberg algorithm. Adjusted p-values < 0.05 were considered significant.

R code used for data analysis has been provided as Supplementary File 1.

### Quantitative reverse-transcribed PCR (qRT-PCR)

Quantitative reverse-transcribed PCR (qRT-PCR) was performed as described previously^26^ using predesigned TaqMan Gene Expression assays or Sybr Green chemistry (Thermo Fisher Scientific). The geometric mean expression levels of *RPLP0* and *UBC* mRNAs were used as normalization factors. Gene expression levels were calculated using the ∆∆Ct method^27^. Results were analyzed using the Mann–Whitney U-test in GraphPad Prism 5 software (GraphPad Software Inc., La Jolla, CA, USA), and p-values < 0.05 were considered significant. The list of Taqman assays and primers is provided in Supplementary Table S1.

### Functional analysis

Functional analyses were conducted in R (version 3.4.1). Gene Set Enrichment Analysis (GSEA) implemented in the gseGO function from clusterProfiler package (version 3.4.4)^28^ was used to link gene expression profiles with Gene Ontology (GO) terms. GO terms were limited to those with between 100 and 300 genes mapped. enrichPathway function from ReactomePA package (version 1.20.2) was used to associate selected gen set with Reactome pathways. Resulting p-values were adjusted for multiple hypothesis testing using the Benjamini–Hochberg algorithm.

## Results

Transcriptome analysis was carried out using samples from patients with two cholestatic liver diseases (ChLDs), PBC and PSC, and two IBDs, CD and UC. While all of these disorders present unique clinicopathological features, ChLDs and IBDs may share underlying processes common to their pathogenesis. Microarray-based assays were conducted by hybridization of 370 RNA samples to Human HT-12 v4 Expression BeadChip microarrays. Of the 370 samples, 90, 45, 95, and 93 were from patients with PBC, PSC, CD, and UC, respectively, while 47 were from healthy controls. Transformation of gene expression variables from each array to their corresponding principal-component scores revealed that the consistency of the microarray data sets was as expected (Supplementary Fig. 1).

The number of differentially expressed genes detected for comparisons of CD and UC with controls were similar (Supplementary Table S2, 4649/4071, respectively). The concordance between the most significantly differentiating genes for both diseases was almost perfect with Spearman correlation coefficient equal 0.93 (Supplementary Fig. 2A) and higher than the correlation between PBC and PSC (Supplementary Fig. 2B). Therefore, while looking for the common functional alterations CD and UC were merged into single IBD group.

Although it is believed that the etiology of early and late onset IBD is different, the whole transcriptome expression pattern haven’t differentiate children and adult patients (Supplementary Fig. 1C,D). Also, the most significant expression differences between each age group and controls were similar (Supplementary Fig. 2C,D).

**Figure 1:**
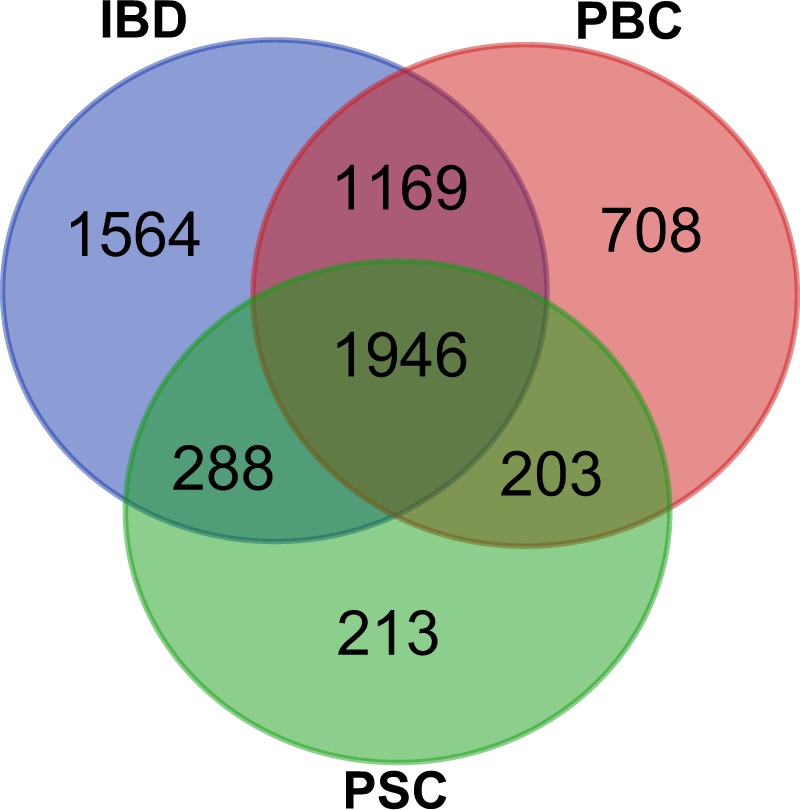
Venn diagrams illustrating the number of differentially expressed transcripts (adjusted p-value < 0.05) in blood samples from patients with PBC, PSC, and IBD compared with those from healthy controls. PBC; Primary biliary cholangitis, PSC; primary sclerosing cholangitis, IBD; inflammatory bowel disease.

According to pair-wise comparisons, 4026, 2650 and 4967 genes were differentially expressed between healthy controls and patients with PBC, PSC, and IBD (combined results of CD and UC), respectively (Fig. 1). Of these, 1946 genes were common to all three comparisons.

### Functional analysis according to GO subcategories

Forty-three GO terms were over-represented among these common probe sets, 23, 12, and 8 of which were attributed to “biological process” (BP), “molecular function” (MF), and “cellular component” (CC) GO terms, respectively (Table 2). The majority of over-represented terms were related to mitochondrial respiration and ATP synthesis, with a few associated with signal transduction by small GTPases and membrane biogenesis and trafficking.

**Table 2:** GO terms over-represented among 1946 probe sets that significantly differentiated disease from control samples in all three comparison groups (i.e., PBC, PSC, and IBD, compared with controls).

ID	Description	qvalue
**Biological process**		
GO:0042773	ATP synthesis coupled electron transport	1.66E-10
GO:0022904	respiratory electron transport chain	1.66E-10
GO:0042775	mitochondrial ATP synthesis coupled electron transport	1.66E-10
GO:0022900	electron transport chain	1.66E-10
GO:1902600	hydrogen ion transmembrane transport	3.4E-08
GO:0070125	mitochondrial translational elongation	4.55E-07
GO:0010257	NADH dehydrogenase complex assembly	5.83E-07
GO:0032981	mitochondrial respiratory chain complex I assembly	5.83E-07
GO:0097031	mitochondrial respiratory chain complex I biogenesis	5.83E-07
GO:0043087	regulation of GTPase activity	6.04E-05
GO:0043624	cellular protein complex disassembly	0.000260
GO:0000956	nuclear-transcribed mRNA catabolic process	0.000294
GO:0022613	ribonucleoprotein complex biogenesis	0.00061
GO:0051056	regulation of small GTPase mediated signal transduction	0.00168
GO:0045047	protein targeting to ER	0.0057
GO:0019080	viral gene expression	0.0099
GO:0072599	establishment of protein localization to endoplasmic reticulum	0.0107
GO:0019083	viral transcription	0.0116
GO:0010608	posttranscriptional regulation of gene expression	0.0265
GO:0002431	Fc receptor mediated stimulatory signaling pathway	0.033
GO:0006906	vesicle fusion	0.037
GO:0043299	leukocyte degranulation	0.043
GO:0044033	multi-organism metabolic process	0.049
**Molecular function**		
GO:0015078	hydrogen ion transmembrane transporter activity	1.72E-09
GO:0003735	structural constituent of ribosome	5.8E-09
GO:0003954	NADH dehydrogenase activity	5.8E-09
GO:0008137	NADH dehydrogenase (ubiquinone) activity	5.8E-09
GO:0050136	NADH dehydrogenase (quinone) activity	5.8E-09
GO:0005096	GTPase activator activity	0.00209
GO:0009055	electron carrier activity	0.0033
GO:0005085	guanyl-nucleotide exchange factor activity	0.0150
GO:0044769	ATPase activity, coupled to transmembrane movement of ions, rotational mechanism	0.0150
GO:0017137	Rab GTPase binding	0.0150
GO:0015399	primary active transmembrane transporter activity	0.032
GO:0015405	P-P-bond-hydrolysis-driven transmembrane transporter activity	0.032
**Cellular component**		
GO:0098800	inner mitochondrial membrane protein complex	6.27E-13
GO:0070469	respiratory chain	4.12E-11
GO:0070069	cytochrome complex	0.00071
GO:0099568	cytoplasmic region	0.00201
GO:0030667	secretory granule membrane	0.0117
GO:0061695	transferase complex, transferring phosphorus-containing groups	0.034
GO:1904115	axon cytoplasm	0.040
GO:0048475	coated membrane	0.048

When the 1946 genes commonly dysregulated in all three disorders were annotated according to the Reactome signaling pathway database, 42 pathways were identified (Supplementary Table 3). Among these, the following terms exhibited the highest level of significance: R-HSA-163200, Respiratory electron transport, ATP synthesis by chemiosmotic coupling, and heat production by uncoupling proteins (adjusted p = 3.01E-17); R-HSA-1428517, The citric acid (TCA) cycle and respiratory electron transport (adjusted p = 1.59E-14); R-HSA-611105, Respiratory electron transport (adjusted p = 4.18E-14); R-HSA-6799198, Complex I biogenesis (adjusted p = 1.17E-09); and R-HSA-5368286, Mitochondrial translation initiation (adjusted p = 4.43E-09).

Next, GSEA was used to link genes differentially expressed in patients with PBC, PSC, CD, and UC compared with healthy controls and GO terms. Altogether, genes differentially expressed between at least one disease and the control group were attributed to 78 BP, 26 MF, and 23 CC terms (Supplementary Table 4). Of these, all (53 PB, 21 MF, and 15) were in ChLDs, while 35 BP, 21 MF, and 13 CFC terms were identified in IBDs.

Terms common to ChLDs included 10 BP, 9 MF, and 13 CC terms, and those shared by IBDs comprised 7 BP, 9 MF, and 3 CC terms. Of these, one BP, six MF, and one CC term were common to all four diseases studied, while four BP, two MF, and four CC terms were common to three diseases (Table 3). The majority of terms, along with their child and synonymous terms, which were enriched for differentially expressed genes in one or two of the diseases studied, were related to the endomembrane system, regulation of membrane dynamics by GTPase-mediated processes, and secretion of proinflammatory molecules.

**Table 3:** GO terms significantly associated with changes in gene expression between control and disease samples according to GSEA analysis.

Biological process					
ID	Description	PBC	PSC	CD	UC
GO:0060627	regulation of vesicle-mediated transport	1	1	1	1
GO:0006906	vesicle fusion	1	1	1	0
GO:0016050	vesicle organization	1	1	0	1
GO:0031346	positive regulation of cell projection organization	1	1	1	0
GO:0032479	regulation of type I interferon production	1	1	1	0
**Molecular function**					
ID	Description	PBC	PSC	CD	MF
GO:0003682	chromatin binding	1	1	1	1
GO:0004674	protein serine/threonine kinase activity	1	1	1	1
GO:0005096	GTPase activator activity	1	1	1	1
GO:0004386	helicase activity	1	1	1	1
GO:0005085	guanyl-nucleotide exchange factor activity	1	1	1	1
GO:0017016	Ras GTPase binding	1	1	1	1
GO:0005543	phospholipid binding	1	1	0	1
GO:0003779	actin binding	1	1	1	0
GO:0022804	active transmembrane transporter activity	1	1	0	1
**Celullar component**					
ID	Description	PBC	PSC	CD	UC
GO:0061695	transferase complex, transferring phosphorus-containing groups	1	1	1	1
GO:0005924	cell-substrate adherens junction	1	1	0	1
GO:0005765	lysosomal membrane	1	1	0	1
GO:0030667	secretory granule membrane	1	1	1	0
GO:0015629	actin cytoskeleton	1	1	1	0

When genes downregulated in blood samples from patients with PBC, PSC, and IBD compared with healthy controls were annotated according to the Reactome database, the following pathways were identified in all three comparisons: R-HSA-163200, Respiratory electron transport, ATP synthesis by chemiosmotic coupling, and heat production by uncoupling proteins; R-HSA-611105, Respiratory electron transport; R-HSA-5389840, Mitochondrial translation elongation; R-HSA-5368286, Mitochondrial translation initiation; R-HSA-5419276, Mitochondrial translation termination; R-HSA-5368287, Mitochondrial translation; R-HSA-1428517, The citric acid (TCA) cycle and respiratory electron transport; and R-HSA-1852241, Organelle biogenesis and maintenance.

### Expression of genes selected for potential use in diagnostic screening

Among the several hundred probe sets differentially expressed between disease and control groups, the majority exhibited relatively low fold-change (FC) differences in expression level, with no FC values exceeding 1.5 (Fig. 1). To determine whether genes differentially expressed in peripheral blood cells could be used for diagnostic screening, we selected 13 (*EMR1*, *IFI27*, *PLCB2*, *RARA*, *SORL*, *STAT1*, *ABCG1*, *C15orf39*, *LYN*, *PLEKHG3*, *ATG2*, *MME*, *DEFA1*), 15 (*MME*, *FOXO3*, *DBI*, *IFI27*, *HSPE1*, *BOLA2*, *ABCG1*, *PLCB2*, *DYSF*, *CLC*, *PRSS33*, *RAP1*, *GAP*, *RNF182*, *RPS28*), and 7 (*OPLAH*, *ALPL*, *SLC26A8*, *PFKFB3*, *MMP25*, *TLR5*, *DYSF*) genes with expression levels significantly altered in patients with PBC, PSC, and IBDs, respectively, compared with healthy controls. Selected genes were those with differences with the highest level of significance and relatively high FC values and were used for analysis in a confirmation study to determine expression levels by qRT-PCR, using the same RNA samples as those used for microarray profiling. Of the 13, 15, and 7 selected genes, the levels of 7, 7, and 3, respectively, were confirmed to differ significantly (adjusted p < 0.05) in samples from patients with PSC, PBC, and IBDs relative to those from healthy control individuals (Table 4).

**Table 4:** Results of confirmation analysis of selected gene expression differences by qRT-PCR. FC, fold-change; AUC, area under the curve.

PSC	p value	FC	AUC	PBC	p value	FC	AUC	IBDs	p value	FC	AUC
MME	**0.00039**	1.3	0.709	EMR1	**1.67E-06**	1.29	0.643	OPLAH	**1.13E-05**	1.55	0.603
FOXO3	**0.000267**	1.5	0.715	IFI27	**1.69E-07**	4.51	0.656	ALPL	**9.34E-09**	1.61	0.650
ABCG1	**2.78E-06**	0.63	0.776	PLCB2	0.003413	1.14	0.587	SLC26A8	**1.01E-10**	1.80	0.568
CLC	**0.000136**	1.97	0.725	RARA	**0.00016**	1.17	0.612	PFKFB3	**0.003463**	1.30	0.601
PRSS33	**0.000311**	2.21	0.715	STAT1	**7.56E-20**	1.42	0.771	MMP25	**1.8E-05**	1.41	0.600
				ABCG1	**1.43E-08**	1.26	0.669	TLR5	**1.73E-10**	1.45	0.648
				C15orf39	**1.26E-05**	1.33	0.630	DYSF	**1.47E-07**	1.53	0.622

Next, we assessed the diagnostic potential of all selected genes using an independently recruited cohort of patients and controls. Replication cohorts included 71 patients with PSC, 292 with PBC, and 727 with IBD, along with 206 (PSC, 37; PBC, 138; IBD, 196) controls. The IBD group consisted of 393 patients with CD (253 children and 140 adults) and 334 with UC (199 children and 135 adults). Pair-wise comparisons of qRT-PCR results revealed statistically significant differences (adjusted p < 0.05) in expression of five, seven, and six genes between the control group and patients with PSC, PBC, and IBDs, respectively (Table 5).

**Table 5:** Results of replication analysis of selected gene expression differences by qRT-PCR using samples from an independent cohort.

Gene	Crohn’s disease			Ulcerative colitis			Active IBDs		
	P value	FC	AUC	P value	FC	AUC	P value	FC	AUC
OPLAH	**5.98E-07**	1.53	0.628	**0.0037**	1.57	0.576	**1.03E-08**	2.49	0.686
ALPL	**5.44E-11**	1.63	0.666	**0.0002**	1.59	0.597	**2.2E-09**	2.25	0.693
SLC26A8	**2.41E-13**	1.93	0.686	**2E-05**	1.66	0.611	**1.47E-12**	2.68	0.728
PFKFB3	**7.2E-05**	1.30	0.601	0.2192	1.30	0.601	**1.21E-09**	1.82	0.696
MMP25	**1.38E-07**	1.45	0.633	0.0162	1.36	0.633	**1.36E-07**	1.86	0.670
TLR5	**6.46E-13**	1.51	0.682	**2E-05**	1.38	0.682	**1.25E-13**	2.06	0.739
DYSF	**7.63E-11**	1.63	0.665	**0.0038**	1.43	0.575	**5.92E-10**	2.06	0.699

FC, fold-change; AUC, area under the curve.

Next, the diagnostic potential of the mRNAs identified as differentially expressed was assessed using receiver operating characteristic (ROC) curves and area under the curve (AUC) analyses. The AUC-ROC values in PSC, PBC, and IBDs were in the ranges 0.709–0.776, 0.587–0.771, and 0.568–0.650, respectively (Table 5). These values indicate that the tested markers have insufficient discriminatory properties to be applicable for clinical practice. Similar analyses were performed for the CU and UC patient subgroups. AUC-ROC values were in the ranges 0.601–0.682 and 0.575–0.682, respectively, despite highly statistically significant differences in mRNA levels between controls and both the CD and UC subgroups. Furthermore, the highest statistically significant differences for the selected transcripts were obtained for comparisons between active IBDs and controls (range, 1.36E-07 to 1.25E-13). Nevertheless, the corresponding AUC-ROC values were only slightly higher (range, 0.670–0.739); therefore, our data do not confirm that assessment of levels of these transcripts has discriminatory power to distinguish between samples from patients with disease and healthy controls, even for patients with active intestinal inflammation.

## Discussion

Crosstalk between the gut and the liver may contribute to common mechanisms underlying liver diseases and gastrointestinal and immune disorders. The gut and liver communicate via the biliary tract, portal vein, and systemic circulation^29^; the liver releases BAs and numerous bioactive mediators, while various metabolites produced in the intestine, by both organisms themselves and their gut microbiota, translocate to the liver through the portal vein.

### Functional analysis of WBC gene expression profiles across PBC, PCS, and IBDs

High-density microarrays allow the measurement of gene expression without prior knowledge of expression profiles. Expression profiles repeatedly measured in whole blood samples from healthy subjects generate repeatable data, from each individual subject, over several months^30^. Specific profiles associated with affected status have been identified in a wide range of diseases, including autoimmune and inflammatory diseases, infectious disorders, psychiatric, cardiovascular, neurological, and neoplastic diseases, and even various environmental factors^31,32^. Among associated environmental factors, blood transcriptome variables could identify associations of socioeconomic status with chronic inflammation^33–35^ and exhibited species- and strain-level specificity in discrimination of viral, bacterial, and eukaryotic infectious diseases, including acute and chronic active Epstein–Barr virus infection and response to tuberculosis treatment^36^. Predictive biomarkers in peripheral blood samples can identify patients with intracranial aneurysms^37^, be used to stratify patients according to disease progression before and after the onset of type 1 diabetes^38–41^, and classify systemic lupus erythematosus and rheumatoid arthritis by prediction of their responsiveness to anti-IFN therapy^42,43^. A few studies have also described alterations of WBC gene expression profiles in IBDs^18–21^.

In this study, we evaluated the molecular alterations underlying PBC, PSC, and IBDs, by functional analysis of microarray data sets through annotation according to the GO and Reactome databases. The majority of terms extracted, based on enrichment for genes differentially expressed in pair-wise comparisons between healthy controls and patients with PBC, PSC, and IBD, shared common profiles related to the vesicle endomembrane system and GTPase-mediated processes. A second major group of GO terms attributed to probe sets with expression changes in all three disease types (PBC, PSC, and IBDs) were related to mitochondrial function. Overall, these terms represent immunological and inflammatory pathways related to cellular stress. Similar functional alterations in WBC transcriptomes were also reported in many of the conditions mentioned above.

Dysregulation of innate and adaptive immune processes is associated with both IBDs and autoimmune fibrous cholangiopathies^6,44–52^. The epithelium of the gastrointestinal tract forms a physical barrier against microbes, and Paneth and goblet cells monitor the bacterial community and regulate host–microbe homeostasis through the production of antimicrobial peptides and mucins. Once the intestinal defense system is affected, or the ecological organization of the healthy gut microbiota is disturbed, immune and inflammatory responses are activated, and can lead to the accumulation of ROS, endoplasmic reticulum (ER) stress, and mitochondrial dysfunction^53,54^. Gut dysbiosis may also be related to alterations in BAs; increased concentrations of hydrophobic BAs may lead to mitochondrial and ER stress-related activation of death receptors and production of inflammatory mediators, such as cytokines, chemokines, and adhesion molecules. Overall, such changes can initiate cholangiocyte cytotoxicity; therefore, the BA–intestinal microbiota–cholestasis triangle is postulated to play a vital role in the pathogenesis of PBC and PSC^44^.

The mechanisms underlying autoimmune liver diseases and gastrointestinal disorders are associated with recirculation of the cell membrane. Exosome vesicles packed with bioactive molecules are involved in cytokine secretion and adaptive immune responses^55,56^ and act as mediators between neighboring cells and distant organs^57,58^. The intracellular transport and delivery of vesicles to the plasma membrane involves GTP-binding proteins^59^ and depends on actin cytoskeleton organization, which dynamically regulates directed endosome traffic and recycling involved in the immune and stress responses^60,61^. Autophagy, an effector mechanism of cellular senescence that blocks the proliferation of cells that harbor genomic injuries, is a lysosome-dependent protective response against various cellular stresses. Autophagy involves degradation and recycling of protein aggregates and damaged organelles and is pivotal for secretion of proteins and production of antimicrobial peptides. The autophagy process regulates a number of cellular functions, including inflammation and adaptive immunity, host defenses, mitochondrial homeostasis, and lipid metabolism, and controls the balance between abnormal immune activation and inflammation^53,54,62–65^.

Finally, the majority of GO nodes extracted from blood transcriptomes were common to phenotypically dissimilar disorders, including ChLDs and IBDs, and were consistent with previous studies uncovering alterations of WBC gene expression in IBDs^18–21^.

### The diagnostic utility of screening for expression of selected WBC genes in PBC, PCS, and IBDs

Gene expression microarray technology can be used to identify genes that are differentially expressed between predefined groups of samples (class comparison), genes whose expression differs across predefined classes of genes (class prediction), and genes that allow classification of molecular subgroups among individuals with seemingly homogenous phenotypes (class discovery). The final results of expression profiling consist of lists of measurements directly linked to genes, some of which may be used as diagnostic, prognostic, or predictive biomarkers. Biomarkers are typically identified by high-throughput methods and subsequently validated by standard molecular methods. In this study, the selection of potential biomarkers was conducted using microarray profiling of gene expression and, since microarray data typically exhibit a low degree of reproducibility^66^, the selected measurements were directly verified by confirmation analysis and indirectly confirmed by qRT-PCR replication studies.

Our microarray-based studies identified thousands of probe sets that differed between disease and control samples; however, the majority of these exhibited low FC values. As higher FC values are positively correlated with the probability that a biomarker can meet the expectations required for clinical utility, we selected genes exhibiting the most statistically significant and largest FC differences between patient and control samples. Although the FC values of the majority of selected genes did not exceed two, both confirmation and replication studies demonstrated that some of them exhibited significant differences in expression between the disease and control groups, with the highest level of significance in patients with active IBDs (p-value range, 1.36E-07 to 1.25E-13). Additionally, we found that 86 differentially expressed genes from our study were common with a set of 133 genes that were designated by Peters *et al*.^67^ as the key driver genes of IBD (Supplementary Fig. S3). Of these 37 were shared among the diseases and 15, 7, and 3 were unique for IBD, PBC and PSC, respectively. This extensive overlap again indicates a functional link between IBD susceptibility genes expression contributing to a discrete systemic inflammation that can be portrayed in blood transcriptome.

Numerous previous studies reported the clinical utility of blood RNA expression profiles; however, many did not perform further validation experiments to demonstrate the utility of their assays for clinical diagnosis. Although medical classification should ideally be binary, i.e., dividing a population by the presence or absence of disease, the majority of molecular biomarkers generate results that overlap between health and disease states. Consequently, most so-called biomarkers can discriminate between groups of patients and controls, rather than being able to consistently and completely distinguish individuals with, from those without, a disease of interest. AUC-ROC values are an appropriate means of assessing the relationship between the sensitivity and specificity of a biomarker across all potential cut-off values. AUC-ROC values >0.8 are assumed to represent moderate (good) discriminatory power, with those >0.9 considered to indicate high (excellent) power to distinguish between analyzed groups. Unexpectedly, according to the AUC-ROCs calculated based on qRT-PCR analysis of expression levels in this study, no single RNA reached diagnostic potential. Our results are consistent with the AUC-ROC values calculated for changes in blood transcriptional levels determined by monitoring UC patients over time in a previous study, which did not exceed 0.8^20^; however, they differ from the results of a recently published study reporting a panel of six genes that could distinguish CD and UC with AUC-ROCs ranging from 0.89 to 0.99^19^. In the latter study, the predictive performance was based on PCR data from only 20 samples^19^. Indeed, blood expression profiles have previously been examined in rather small populations, and analyses of differentially expressed genes have generally produced results with overlap between healthy and diseased samples^18–21^. Our microarray screening, followed by confirmatory qRT-PCR studies, was conducted using 370 RNA samples, and several hundred additional samples were included in the replication analysis. Therefore, the results of our investigation can be considered reliable, since the approach applied was appropriate for a search for new biomarkers and employed a relatively large patient population.

To summarize, although we are witnessing the era of molecular diagnostics, of the numerous potential biomarkers identified by high-throughput methods in chronic autoimmune diseases, none has proven ideal to date^68,69^. This study indicates that microarray-based profiling of blood gene expression levels can support research into the molecular mechanisms underlying disease, while being less useful for the selection of diagnostic biomarkers for use in clinical practice.

## Supplementary information

Supplementary figures

Table S1

Table S2

Table S3

Table S4

Supplementary file 1

## Data Availability

The results of microarray measurements have been deposited in Gene Expression Omnibus database, entry GSE119600.
