# Supplementary material for: Common functional alterations identified in blood transcriptome of autoimmune cholestatic liver and inflammatory bowel diseases
Source: Sci Rep. 2019 May 10;9:7190. doi: 10.1038/s41598-019-43699-1 (PMC6510750; doi:10.1038/s41598-019-43699-1)
Supplement: Supplementary file 1 — Supplementary figures [file 41598_2019_43699_MOESM1_ESM.pdf]

Common functional alterations identified in blood transcriptome of autoimmune cholestatic liver and inflammatory bowel diseases

Jerzy Ostrowski[1,2,], Krzysztof Goryca[1], Izabella Lazowska[3], Agnieszka Rogowska[2], Agnieszka Paziewska[2], Michalina Dabrowska[1], Filip Ambrozkiewicz[1], Jakub Karczmarski[1], Aneta Balabas[1,] Anna Kluska[1], Magdalena Piatkowska[1], Natalia Zeber-Lubecka[2], Maria Kulecka[2], Andrzej Habior[2], Michal Mikula[1], The Polish IBD study Group, The Polish PBC study Group

[1]Department of Genetics, Maria Sklodowska-Curie Institute – Oncology Centre, Warsaw 02-781, Poland; [2]Department of Gastroenterology and Hepatology, Medical Center for Postgraduate Education, Warsaw 02-781; [3]Department of Pediatric Gastroenterology and Nutrition, Medical University of Warsaw, Warsaw 02-091, Poland;

***Correspondence:** Jerzy Ostrowski, MD, PhD; Cancer Center-Institute, Roentgena 5, 02-781 Warsaw, Poland, Tel: +48 225462575, E-mail: jostrow@warman.com.pl

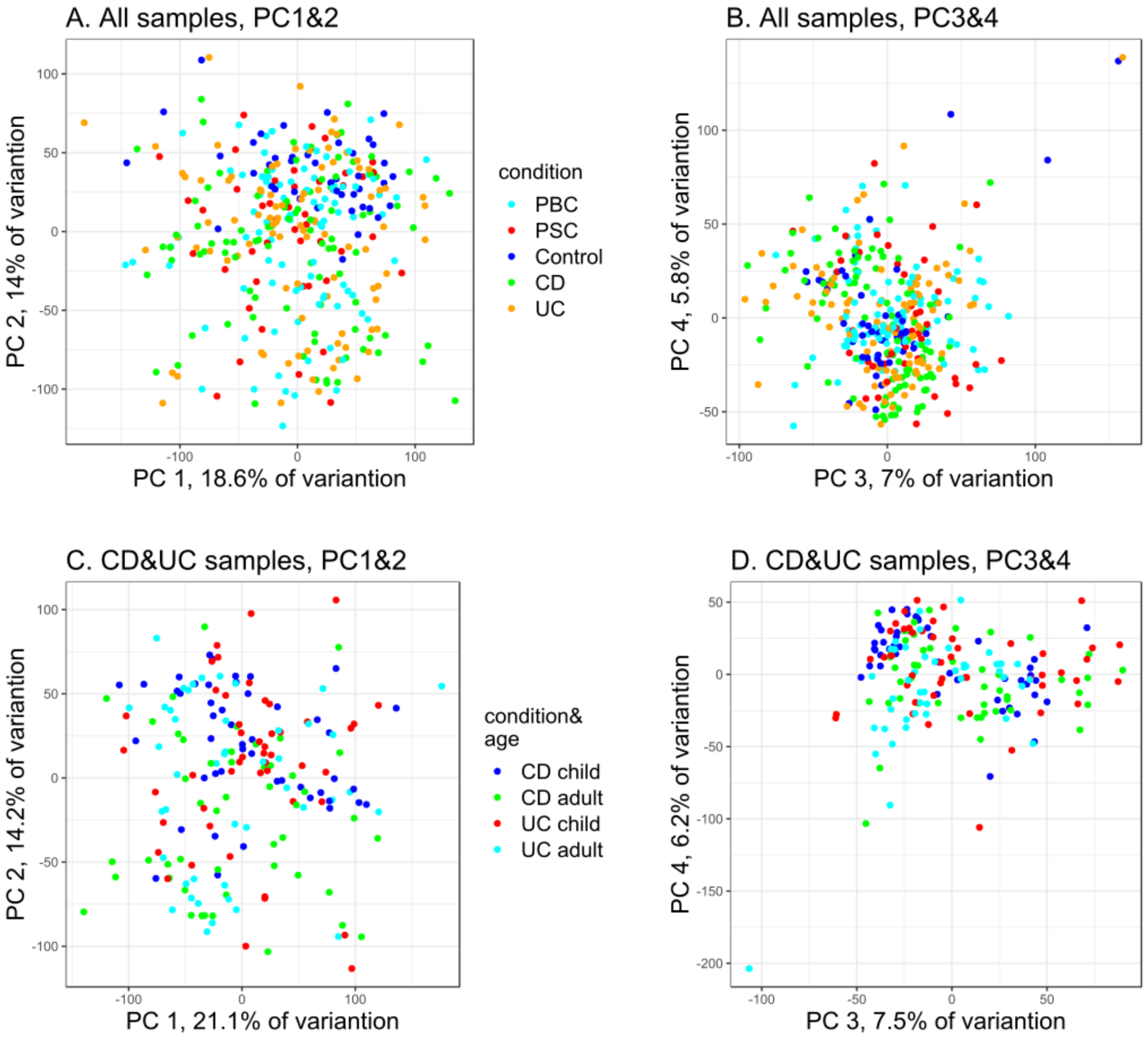

Figure S1. PCA results for microarray expression values. Plots of the first vs the second principal component (panels A,C) and the third vs the fourth principal component (panels B,D). Result for all samples (panels A,B) and IBD samples only (panels C,D).

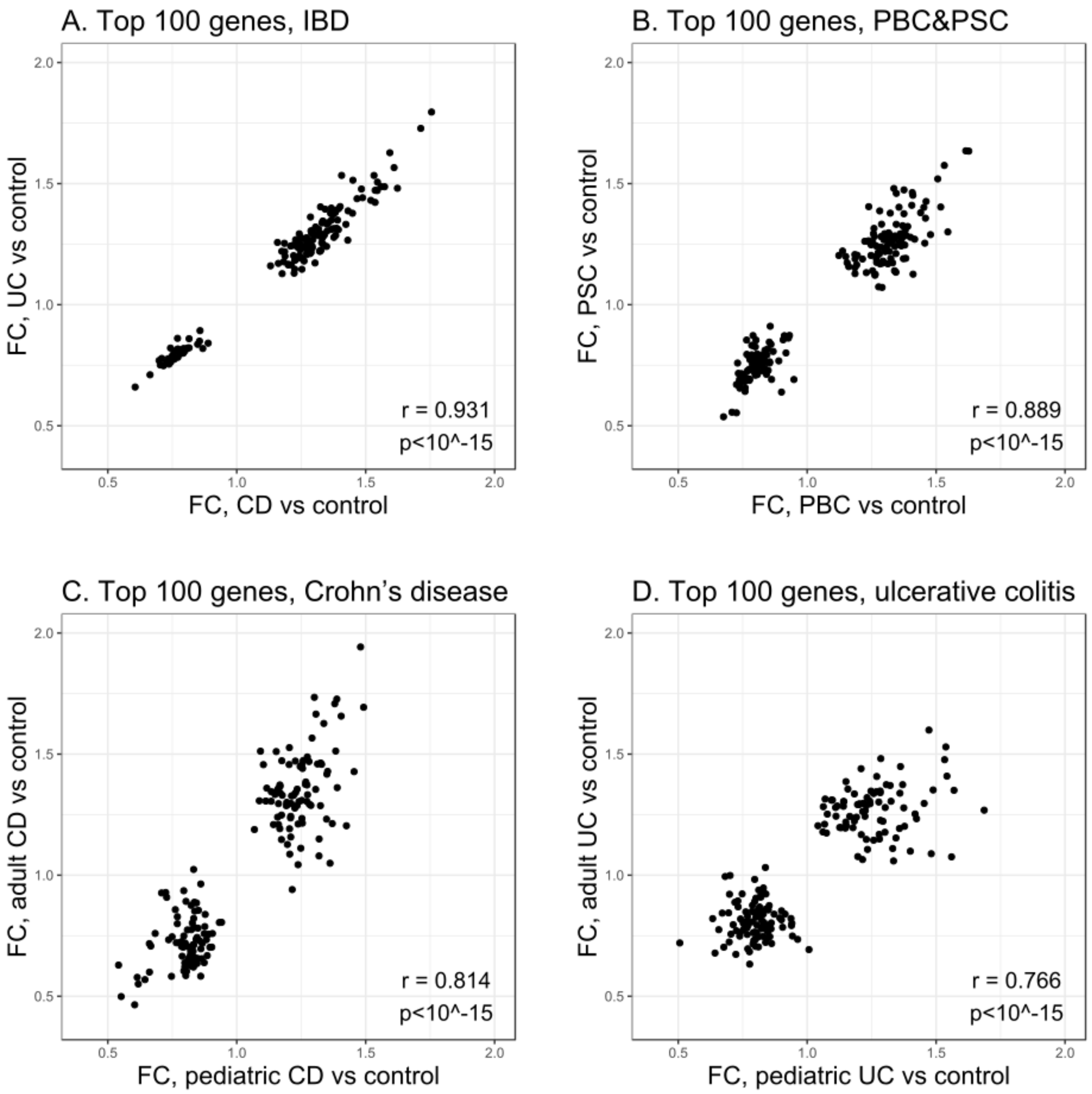

Figure S2. Correlation of FC values in comparisons of selected conditions vs control samples. For each plot the top 100 genes (according to p-value) from appropriate comparisons are selected. Spearman correlation coefficient and corresponding p-value are given in the bottom right corner of each panel.

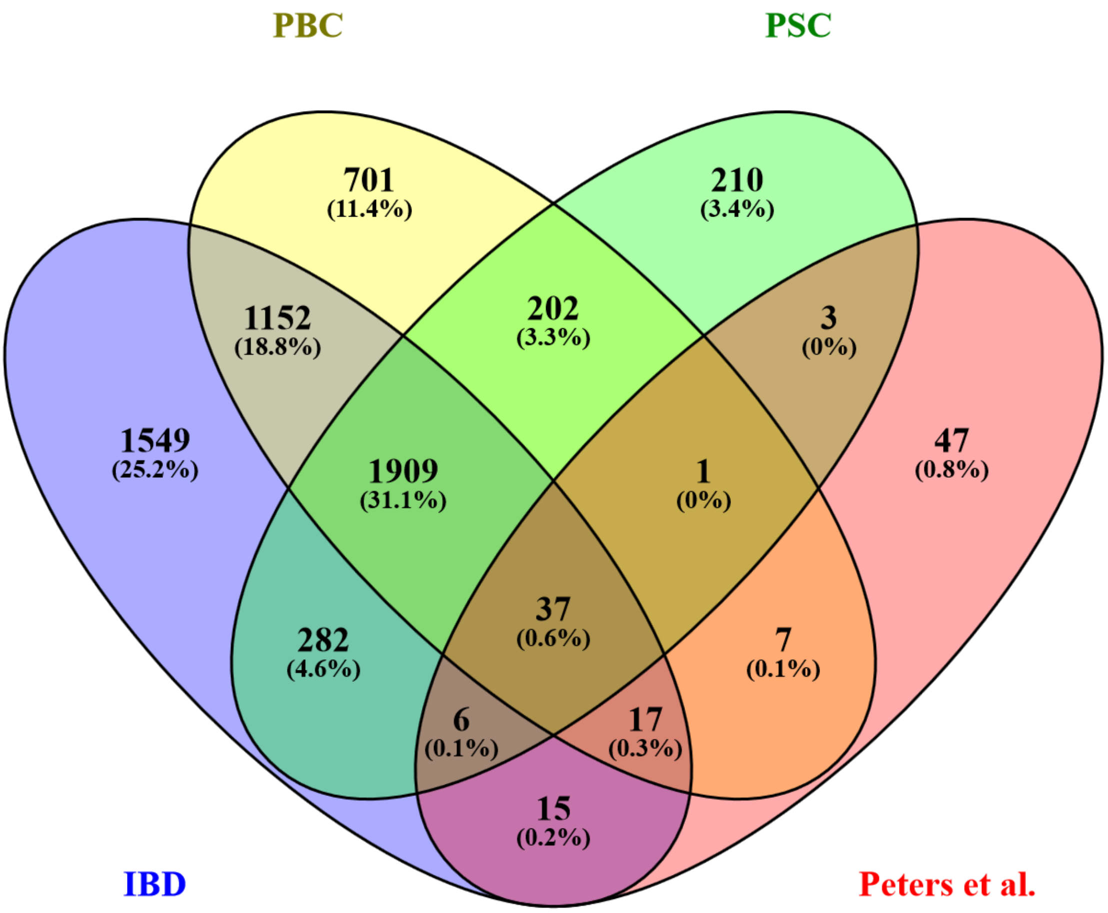

Figure S3. Intersection of gene sets differentiating each given condition (PBC, PSC, IBD) from control samples and set of genes associated with IBD according to Peters et al.
